# When Patient and Clinician Miss the Signal: A Delayed Diagnosis of Pancoast Tumor

**DOI:** 10.7759/cureus.91732

**Published:** 2025-09-06

**Authors:** Leila Laouar, Nadia Dammene Debbih, Narriman Laouar

**Affiliations:** 1 Department of Pulmonology, Mustapha University Hospital Center, Algiers, DZA; 2 Faculty of Medicine, Youcef El Khatib University of Health Sciences, Algiers, DZA; 3 Department of Cardiology, Mustapha University Hospital Center, Algiers, DZA; 4 Faculty of Medicine, Frères Mentouri Constantine 1 University, Constantine, DZA; 5 Department of Medical Oncology, Benbadis University Hospital Center, Constantine, DZA

**Keywords:** adenocarcinoma, cognitive bias, diagnostic delay, pancoast tumor, shoulder pain, thoracic ct, tobacco use

## Abstract

Pancoast tumors, located at the pulmonary apex, present a diagnostic challenge due to their atypical clinical presentation, often mimicking benign musculoskeletal conditions of the shoulder. We report the case of a 49-year-old man with a history of chronic tobacco use who presented with persistent right shoulder pain, initially attributed to rotator cuff tendinopathy based on suggestive ultrasonographic findings. Despite symptom progression and nocturnal pain refractory to conventional therapy, no early thoracic imaging was pursued. A previously performed shoulder radiograph showed apical pleural thickening that went unrecognized by a non-specialist, missing an early sign of malignancy. Diagnostic delay was further compounded by the patient’s reluctance to undergo chest imaging, fueled by fear of a cancer diagnosis and the psychological burden associated with long-term smoking. The diagnosis of apical bronchogenic adenocarcinoma was delayed until an advanced stage, precluding curative treatment. The patient died 12 days after diagnosis from cachexia and sepsis. This case highlights the need for the early consideration of apical lung tumors in patients with atypical shoulder pain, particularly among smokers. The careful interpretation of initial imaging and the awareness of psychological barriers to diagnosis are critical to improving outcomes.

## Introduction

Pancoast tumors are rare lung cancers, characterized by the early invasion of adjacent anatomical structures, including the brachial plexus, first ribs, cervical lymph node chains, and subclavian vessels. This pattern of invasion underlies their classification as T3 (the invasion of the chest wall, parietal pleura, or sympathetic chain) or T4 (the invasion of the brachial plexus, vertebral body, or major mediastinal vessels), depending on the extent of locoregional spread [1]. Because of their apical and peripheral anatomical position, respiratory symptoms are typically absent at onset. Instead, the clinical picture is often dominated by cervicobrachial pain, frequently misdiagnosed as benign musculoskeletal disorders (tendinopathy, cervical radiculopathy, rotator cuff disease, and degenerative spine disorders) [2]. This deceptive presentation contributes to frequent diagnostic delays, particularly among chronic smokers. Multiple studies underscore the importance of a thorough initial evaluation in cases of unexplained radicular pain. Prognosis depends on early recognition, rapid thoracic imaging, histopathologic confirmation, and a multimodal therapeutic strategy combining chemotherapy, radiotherapy, and surgical resection. This case highlights the diagnostic pitfalls associated with Pancoast-Tobias tumors and the clinical consequences of delayed diagnosis on treatment options and overall prognosis.

## Case presentation

A 49-year-old man, an active smoker (20 cigarettes/day for 18 years) and occasional cannabis user (one joint/week), with a medical history of right elbow tendinopathy treated with nonsteroidal anti-inflammatory drugs (NSAIDs) and physical therapy, presented with chronic right shoulder pain progressing over eight months. The pain was non-mechanical, not exacerbated by movement, and was initially attributed to a recurrence of tendinopathy. This hypothesis was reinforced by an ultrasound that suggested benign musculoskeletal involvement. Despite treatment with NSAIDs, corticosteroid injections, and physiotherapy, the pain progressively intensified, became nocturnal, and was resistant to standard analgesics.

Physical examination

Physical examination revealed a performance status (PS) of three, tachypnea (25 breaths/minute), oxygen saturation of 93%, a body temperature of 38.2°C, and moderate tachycardia (110 beats per minute {bpm}). Blood pressure was 100/50 mmHg. BMI was 16.2 kg/m², indicating severe malnutrition. Right shoulder pain was lancinating and radiated to the upper limb, with maximal tenderness over the deltoid region and adjacent thoracic wall. No Horner syndrome was observed. Neurologic examination revealed right upper limb weakness without clear sensory deficits. The percussion of the right hemithorax elicited painful dullness. No supraclavicular or cervical lymphadenopathy or digital clubbing was present.

Laboratory tests

Laboratory analysis revealed marked neutrophilic leukocytosis (white blood cell count: 28.3 × 10⁹/L, 81% neutrophils), elevated C-reactive protein (CRP, 238 mg/L), anemia (hemoglobin: 9.8 g/dL), thrombocytosis (platelet count: 554 × 10⁹/L), hyperglycemia (blood glucose: 1.26 g/L), hyponatremia (sodium: 128 mmol/L), normal potassium levels, and borderline renal clearance (62 mL/minute). Serum albumin was low at 28 g/L. Arterial blood gas analysis showed hypoxemia with evidence of tissue hypoperfusion (pH, 7.38; PaCO₂, 45 mmHg {6 kPa}; PaO₂, 72 mmHg {9.6 kPa}; bicarbonate {HCO₃⁻}, 25 mmol/L; arterial oxygen saturation {SaO₂}, 93%; lactate, 3.5 mmol/L).

Thoracic imaging

**Figure 1:**
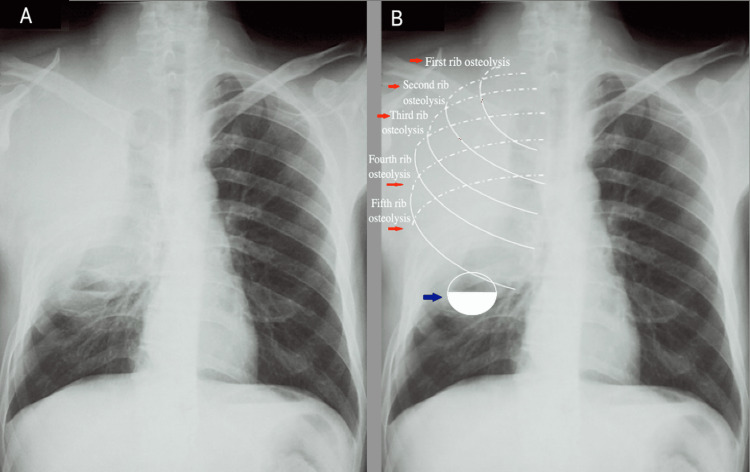
A hypodense focus with an air-fluid interface, consistent with an abscess, is seen at the lower border of the opacity (blue arrow) (A) Frontal chest radiograph showing a dense, homogeneous opacity occupying the upper two-thirds of the right hemithorax, with the lysis of the first to fifth ribs and an air-fluid level at its lower margin. (B) Magnified view of the same radiograph with the schematic reconstruction of the posterior-to-anterior arcs of the first to fifth right ribs (outlined in white and indicated by red arrows), highlighting the extent of rib lysis.

**Figure 2:**
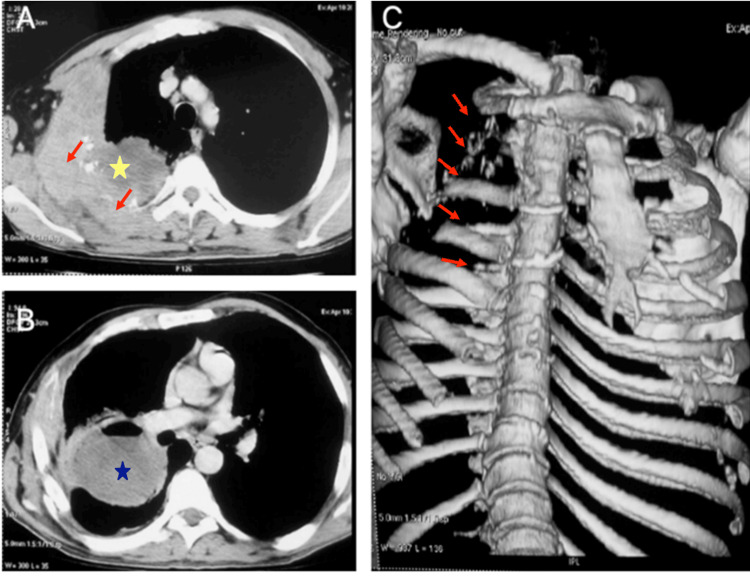
Chest computed tomography. Axial images in the mediastinal window (panels A and B). Panel A shows a peripheral right upper lobe mass (yellow asterisk) with direct chest wall invasion, involving adjacent soft tissues and the arcs of the first through fifth ribs (red arrows). Panel B demonstrates central cavitation within the mass (blue asterisk), suggestive of tumor necrosis or superimposed infection. A three-dimensional bone reconstruction (panel C) depicts the lytic destruction of the right first through fifth ribs (red arrows). Tumor stage at the time of imaging was T4N0Mx.

Chest imaging was delayed until clinical deterioration required reevaluation. Chest radiography revealed a dense, homogeneous opacity occupying the upper two-thirds of the right hemithorax, associated with the lytic destruction of the posterior aspects of the first five ribs and a basal air-fluid level, suggesting parenchymal suppuration (Figure 1). The high-resolution computed tomography (CT) of the chest demonstrated a 12-cm right apical mass with necrosis and cavitation, raising suspicion of superimposed abscess formation. The mass extensively invaded the chest wall, involving the first to fifth ribs, the second thoracic vertebra, and adjacent soft tissues. Additional findings included 2.6-cm hilar lymphadenopathy and a small right pleural effusion, with no evidence of distant metastases at that stage (Figure 2).

Histological diagnosis

Bronchoscopic evaluation revealed the infiltration of the right upper lobe bronchial mucosa. Biopsies confirmed a primary pulmonary adenocarcinoma. Immunohistochemistry demonstrated transcription factor-1 (TTF-1) and cytokeratin 7 (CK7) positivity, consistent with a lung origin, and ruled out epidermal growth factor receptor (EGFR) and anaplastic lymphoma kinase (ALK) mutations.

Management

The patient was promptly initiated on oral morphine for pain control along with empirical antibiotic therapy. Despite these measures, his clinical condition rapidly deteriorated, precluding further diagnostic staging or therapeutic planning. He died 12 days after diagnosis in the setting of profound cachexia and sepsis.

## Discussion

Pancoast tumors predominantly affect men around the age of 60, with a higher incidence in the right lung (59%) and a near-universal association with tobacco use [3]. Our patient matched this profile, with the exception of his relatively young age. In 96% of cases, the initial symptom is neuropathic cervicobrachial pain, reflecting the early invasion of the brachial plexus, particularly the C8-T1 nerve roots [3]. The compression or erosion of osseous structures further exacerbates the pain. These symptoms are typically deep, constant, and refractory to conventional analgesics and radiate along the brachial plexus into the upper limb. Additional neurologic signs may include Horner syndrome (ptosis, miosis, and anhidrosis) [4], hand weakness or atrophy of intrinsic muscles, and paresthesias in approximately 40% of cases [3,5]. Unlike mechanical pain, this pain is often nocturnal and non-mechanical and persists at rest [6]. Pulmonary symptoms such as dyspnea, cough, or hemoptysis usually occur only at advanced stages of the disease [7]. This was consistent with our patient, whose initial presentation consisted of right shoulder pain unrelated to movement, later becoming nocturnal and intense, with the emergence of a productive cough only at a more advanced stage. A diagnosis of tendinopathy was initially made based on an ultrasound that showed findings consistent with a benign musculoskeletal lesion (Figure 1). This misleading presentation led to conventional treatment, NSAIDs, corticosteroid injections, physiotherapy, and acupuncture with no clinical improvement. Such diagnostic delays are common in early presentations of Pancoast tumors, where shoulder pain precedes thoracic or neurologic symptoms. This clinical pitfall has been well documented. Berntheizel et al. reported cases in which patients initially consulted non-specialists, such as chiropractors, for radicular or musculoskeletal symptoms before a thoracic imaging study revealed the neoplastic nature of the condition [8]. Similarly, Kočan et al. emphasized that persistent, severe pain should raise suspicion of an apical tumor [9].

In our case, although chest radiography was prescribed upon symptom aggravation, the patient, an active smoker, delayed the examination. This refusal was driven by a combination of internalized guilt and denial, mechanisms frequently observed in lung cancer patients who are aware of their tobacco exposure. Studies show that current or former smokers with lung cancer often report strong feelings of shame, social stigma, and self-blame, which negatively affect their willingness to undergo screening or diagnostic evaluation [10]. These psychological factors may delay medical consultation and compromise clinical surveillance, as also noted by Carter-Harris et al., who identified perceived smoking-related stigma as a barrier to early detection [11]. Hamann et al. further reported that smokers may engage in avoidance behavior, preferring to ignore alarming symptoms to avoid facing a possible cancer diagnosis [12].

In such contexts, the clinical presentation is often misleading, with shoulder or neck pain being initially attributed to rheumatologic conditions such as bursitis, osteoarthritis, or tendinopathy, as was the case with our patient. This diagnostic confusion leads to a mean delay of 5-10 months [13]; in our case, the delay was eight months. The situation was further worsened by maladaptive self-medication: the patient tripled his cannabis consumption in response to worsening, sleep-disrupting pain, instead of following through with the recommended thoracic imaging.

The radiological diagnosis of Pancoast tumors remains challenging, primarily due to their apical location, which is often poorly visualized on standard chest radiographs. In early stages, chest X-rays may appear normal or show only subtle apical pleural thickening, frequently misinterpreted as benign. However, such findings should raise suspicion when they are irregular, asymmetric, and associated with persistent pain, as highlighted by Panagopoulos et al. [14]. In more advanced stages, chest radiography may reveal more specific features, including apical opacities, the erosion of the upper ribs, or periosteal reactions, as reported by Dartevelle and Macchiarini [15]. In our case, the initial chest X-ray showed a right apical pleural thickening that was considered nonspecific and thus overlooked, although it likely represented the earliest radiological sign of an underlying malignancy. Computed tomography (CT) is the reference imaging modality, allowing the detailed assessment of local tumor extension to paravertebral tissues, the chest wall, the brachial plexus, subclavian vessels, and mediastinal lymph nodes. It also identifies pulmonary cavitation and distant metastases [16]. Magnetic resonance imaging (MRI) plays a complementary role, particularly in delineating brachial plexus involvement and assessing the tumor’s relationship with surrounding neurovascular structures. It is considered essential in the preoperative staging of Pancoast tumors.

The diagnosis of Pancoast tumors is based on bronchial or transthoracic histology, often supported by immunohistochemistry. Tissue samples are typically obtained via transbronchial biopsy or, more commonly, CT- or ultrasound-guided transthoracic needle biopsy. These tumors are predominantly malignant, with adenocarcinoma being the most common histological subtype (50%), followed by squamous cell carcinoma (38%), large-cell carcinoma (6%), and neuroendocrine carcinoma (3%) [15]. Immunohistochemical staining for TTF-1 and CK7 confirms pulmonary origin. In the present case, histology revealed a primary bronchogenic adenocarcinoma without EGFR or ALK mutations, thus indicating standard management without molecular-targeted therapy.

The optimal management of Pancoast tumors relies on a multidisciplinary, trimodal approach, combining chemotherapy, radiotherapy, and surgery. In advanced stages, treatment is palliative, focused on controlling severe neuropathic pain following the WHO analgesic ladder, with the use of opioids, palliative radiotherapy, and, in refractory cases, invasive techniques such as cervical DREZotomy [17]. Prognosis is influenced by vascular, vertebral, or nodal invasion; the presence of Claude Bernard-Horner syndrome; and the completeness of tumor resection [18]. It remains poor in the setting of delayed diagnosis. Reported overall survival rates at two, five, and 10 years are 67.9%, 50.1%, and 31.8%, respectively [3]. This favorable prognosis could not be achieved in our patient due to a major diagnostic delay, complicated by probable sarcopenia and severe pulmonary infection. The rapid progression to infectious cachexia highlights the critical need for early recognition, especially in high-risk populations such as chronic smokers [19].

## Conclusions

Pancoast tumors remain a diagnostic challenge due to their deceptive initial presentation, often mistaken for benign shoulder disorders. Clinicians, particularly those managing shoulder pain, should maintain a high index of suspicion when evaluating persistent cervicobrachial pain, especially in smokers or other high-risk patients. Early and comprehensive neurologic assessment, combined with prompt thoracic imaging, is essential to minimize diagnostic delays. Moreover, addressing psychological barriers, such as denial, stigma associated with smoking, and self-medication, should guide the clinical approach to facilitate timely evaluation. Early referral to a multidisciplinary team, including pulmonology, radiology, oncology, pain management, and psychological support, is crucial to optimize therapeutic options, improve survival, and preserve quality of life. Implementing these recommendations can help reduce diagnostic delays and improve clinical outcomes in patients with Pancoast tumors.
